# Mathematical Modeling and Artificial Intelligence to Explore Connections Between Glaucoma and the Gut Microbiome

**DOI:** 10.3390/medicina61020343

**Published:** 2025-02-14

**Authors:** Madeline C. Rocks, Priyanka Bhatnagar, Alice Verticchio Vercellin, Lorenzo Sala, Brent Siesky, Gal Antman, Keren Wood, Riccardo Sacco, Alon Harris

**Affiliations:** 1 George Washington University School of Medicine and Health Sciences, Washington, DC 20052, USA; mrocks@gwu.edu (M.C.R.); pbhatnagar23@gwu.edu (P.B.); 2 Department of Ophthalmology, Icahn School of Medicine at Mount Sinai, New York, NY 10029, USA; alice.verticchio@mssm.edu (A.V.V.); brent.siesky@mssm.edu (B.S.); antmangal@gmail.com (G.A.); keren.woodshalem@mssm.edu (K.W.); riccardo.sacco@mssm.edu (R.S.); 3 Université Paris-Saclay, INRAE, MaIAGE, 78350 Jouy-en-Josas, France; lorenzo.sala@inrae.fr; 4 Faculty of Medicine, Tel Aviv University, Tel Aviv 69978, Israel; 5 Department of Ophthalmology, Rabin Medical Center, Petah Tikva 4941492, Israel

**Keywords:** glaucoma, microbiome, therapeutic targets, mathematical modeling, artificial intelligence, machine learning, data science

## Abstract

*Background and Objectives:* Glaucoma is a major cause of irreversible blindness, with primary open-angle glaucoma (POAG) being the most prevalent form. While elevated intraocular pressure (IOP) is a well-known risk factor for POAG, emerging evidence suggests that the human gut microbiome may also play a role in the disease. This review synthesizes current findings on the relationship between gut microbiome and glaucoma, with a focus on mathematical modeling and artificial intelligence (AI) approaches to uncover key insights. *Materials and Methods:* A comprehensive literature search was conducted using PubMed and Google Scholar, covering studies from its inception to 1 August 2024. Selected studies included basic science, observational research, and those incorporating mathematical-related models. *Results*: Traditional statistical and machine learning approaches, such as random forest regression and Mendelian randomization, have identified associations between specific microbiota and POAG features. These findings highlight the potential of AI to explore complex, nonlinear interactions in the gut–eye axis. However, limitations include variability in study designs and a lack of integrative, mechanistic models. *Conclusions:* Preliminary evidence supports the existence of a gut–eye axis influencing POAG disease. Combining data-driven and mechanism-driven models with AI could identify therapeutic targets and novel biomarkers. Future research should prioritize longitudinal studies in diverse populations and integrate physiological data to improve model accuracy and clinical relevance. Furthermore, physics-based models could deepen our mechanistic understanding of the gut–eye axis in glaucoma, advancing beyond associative findings to actionable insights.

## 1. Introduction

Glaucoma is a leading cause of irreversible blindness worldwide, with the most common form being primary open-angle glaucoma (POAG) [1]. POAG is a progressive optic neuropathy characterized by loss of retinal ganglion cells (RGCs) and visual field defects [2]. While elevated intraocular pressure (IOP) is a key risk factor, other factors, including systemic inflammatory processes, have been increasingly implicated in the disease’s pathogenesis [2,3,4]. Of particular interest is the human microbiome, which has been linked to other neurodegenerative diseases such as Alzheimer’s and Parkinson’s disease [5,6]. As glaucoma is inherently a neurodegenerative condition, the microbiome’s potential influence on POAG warrants further investigation.

Mechanistically, dysbiosis of the gut, oral, and ocular microbiomes have all been proposed as possible contributors to immune-mediated inflammatory diseases and neurodegenerative conditions through the lipopolysaccharide toll-like receptor 4 (LPS-TLR4) pathway [7]. Bacterial translocation increases LPS products, which trigger proinflammatory responses and mediate local immune activation [8]. TLR4, a bacterial toxin shared between the ocular environment and the gut, responds to LPS by initiating an adaptive immune response and endotoxin pathway that propagates neurodegeneration [7]. A recent review by Hernández-Zulueta et al. identified 25 microbes present in the gut, mouth, or eye linked to glaucoma pathogenesis, emphasizing the relevance of the microbiome in the disease context [9].

This article reviews the current literature exploring the relationship between the human gut microbiome and the development and progression of POAG. We highlight recent studies that utilize mathematical modeling, particularly those enhanced by artificial intelligence (AI) based tools, for their ability to navigate the complexities of the gut–eye axis. The discussion also highlights the potential of underutilized physics-based, mechanism-driven models to provide causal insights and deepen our understanding of the microbiome’s role in POAG pathogenesis.

## 2. Materials and Methods

PubMed and Google Scholar databases were searched to identify the literature from inception through 1 August 2024, exploring relationships between glaucoma and the human microbiome. The initial search included: (“glaucoma” OR “primary open-angle glaucoma” OR “open-angle glaucoma” [Mesh]) AND (“human microbiome” OR “microbiome” OR “microbiota” OR “gut–eye axis” [Mesh]). A subsample search of resulting articles included: (“mathematical modeling” OR “artificial intelligence” OR “mathematics” [Mesh]). Special attention was given to the mathematical journals covering biomedical applications, such as the *Journal of Mathematical Biology*, *Journal of the Royal Society Interface*, and *Mathematical Biosciences and Engineering*. Reference lists from relevant review articles were also examined and cross-referenced. Titles and abstracts of 68 articles were screened by three authors to assess relevancy. A full-text review was conducted for 23 articles identified as relevant. Ultimately, 10 key articles were selected, focusing on studies that demonstrated how AI and mathematical modeling could be used to investigate the impact of the microbiome on glaucoma. These articles included basic science studies, observational clinical studies, and those employing mathematical modeling approaches. Only articles that were written in English, presented original data, discussed POAG and the human microbiome, and utilized mathematical modeling were included. Data were organized using Microsoft Word and Excel.

Potential biases in the selected studies were considered when interpreting findings. Variability in study design (e.g., sample size, population demographics, microbiome sampling techniques, statistical methodologies) may introduce heterogeneity, limiting the generalizability of findings. Additionally, publication bias favoring significant associations may overemphasize certain relationships while underrepresenting null results.

For clarity, we categorized mathematical modeling approaches into two primary classes: (1) data-driven models and (2) mechanism-driven models. Data-driven models rely on empirical data to identify patterns, correlations, and associations within large datasets. These models include classical statistical approaches, such as regression analysis (for modeling relationships between variables) and Principal Component Analysis (PCA, for dimensionality reduction). More advanced methods, such as machine learning (ML) techniques and Mendelian Randomization (MR) also fall under this category. ML techniques include random forest for classification and deep learning for complex pattern recognition. MR, a statistical method used for causal inference, leverages genetic variants as instrumental variables to infer causal relationships between exposures (e.g., gut microbial composition) and outcomes (e.g., glaucoma progression). By using the random allocation of genetic variants at conception, MR minimizes confounding factors and biases, providing a robust framework for causal inferences in observational data.

By leveraging the random allocation of genetic variants at conception, MR minimizes confounding factors and bias, providing a robust framework for establishing causal inferences in observational data. All in all, AI-based methods are considered a subset of the data-driven framework, as they enhance model performance by automating pattern recognition, optimizing predictions, and handling high-dimensional data.

Mechanism-driven (or physics-based) models simulate system behavior using fundamental biological and physical principles. These models are based on equations derived from physical laws, such as fluid dynamics, solute transport, or immune response mechanisms, to provide causal explanations and predictive capabilities.

**Figure 1:**
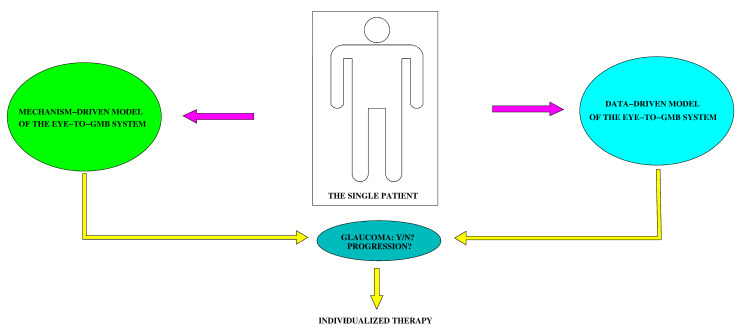
Conceptual scheme representing the use of data-driven and mechanism-driven models to investigate the connection between glaucoma and the gut microbiome (GMB). Arrows in purple color indicate input data provided to each model on the basis of information available about the single patient. Arrows in yellow color indicate the output returned from each model. The information provided by the two outputs may be used as a supporting tool to design an optimal individualized therapy.

A graphical illustration of the use of data-driven and mechanistic models in the study of the connection between glaucoma and gut microbiome (GMB) is given in Figure 1. The conceptual scheme helps understand how information available from the single patient’s clinical history may be provided as input for each model (purple arrows) and how the output returned from each model (yellow arrows) may be used to ascertain the onset of glaucoma and the level of its progression and to help optimize the design of individualized therapy for the cure of the disease.

AI applications are increasingly being explored as computational tools to improve mechanism-driven models. By optimizing simulation parameters, integrating multi-scale datasets, and improving computational efficiency, AI techniques can increase the predictive power and robustness of physics-based approaches. This distinction between data-driven and mechanism-driven models allows for a clearer interpretation of the literature. AI serves both as an enhancer of data-driven frameworks and a computational bridge to optimize mechanistic simulations. Combining these approaches could offer a unified, multi-faceted understanding of the gut–eye axis.

## 3. Results

Most research on the relationship between glaucoma and the human gut microbiome comes from gene marker analysis and RNA sequencing of bacteria, which mainly identify microbiome composition. These studies are limited by the complexity of interactions between the gut microbiome and glaucoma, as well as the challenges of conducting large-scale clinical trials. Data-driven models, including statistical approaches and ML methodologies, offer advantages in analyzing large-scale data from animal or human hosts to identify associations between microbiome features and disease features [10,11]. Studying the effects of microbiomes in clinical settings is challenging due to the complexity of the microbial communities in our bodies. As a result, the development of AI-based tools to enhance data-driven models has emerged as a promising direction for future research. Below, we discuss current evidence of microbiota involvement in POAG, and how data-driven models and other mathematical approaches can address existing data challenges.

Chaiwiang et al. provide a foundational perspective on the role of gut microbiota in diseases such as POAG, highlighting areas where modeling could advance our understanding [11]. Although this study does not utilize mathematical modeling, it emphasizes the potential of such approaches, particularly in bioinformatics and epidemiological modeling, to clarify the complex pathways linking gut dysbiosis to glaucoma. The authors suggest that the gut microbiome may contribute to neuroinflammatory and autoimmune pathways affecting the retina, similar to mechanisms found in other neurodegenerative disorders. This research underscores the need for mathematical modeling to enhance our understanding of the gut–retina axis and suggests that modeling immune-mediated pathways may reveal new insights into POAG.

The relevance of data-driven approaches in microbiome-glaucoma research is demonstrated by studies from Chen et al., Parker et al., Zysset-Burri et al., Vergroesen et al., and Astafurov et al., who apply classical statistical methods to examine correlations between microbial profiles and glaucoma progression [12,13,14,15,16] [Table 1]. Chen et al. used correlation analysis and ANOVA, a method for analyzing the variance across groups, to explore genetic markers associated with POAG, identifying immune-regulatory genes that may serve as biomarkers [12]. While these methods reveal important associations, they are limited in capturing the high-dimensional interactions required to fully understand glaucoma pathogenesis. Similarly, Parker et al. applied PCA and regression techniques to investigate shifts in gut microbiome composition related to POAG, showing how microbial profiles correlate with immune modulations that may influence disease progression [13]. Zysset-Burri et al. focused on the complement system, identifying associations between microbial taxa and immune responses in glaucoma patients, while Vergroesen’s research utilized correlation analysis to highlight microbiome markers that correlate with specific POAG phenotypes [14]. Despite these insights, these studies collectively acknowledge that traditional statistical methods, while valuable for identifying associations, are insufficient for capturing the dynamic, non-linear relationships within the gut–eye axis. Astafurov et al.’s foundational study on the role of microbial taxa in POAG also calls for advanced modeling approaches to transform these associations into clinically actionable insights [16].

In recent years, advanced data-driven models enhanced by AI-based computational tools have been increasingly applied to microbiome studies on POAG, as demonstrated by studies from Yoon et al., Li et al., Wu et al., and Zhou et al. [10,17,18,19] [Table 2]. Yoon and colleagues used random forest regression and rule-mining algorithms to analyze microbiome datasets, identifying Lactococcus as a potential biomarker for POAG severity [17]. This study highlights how ML can uncover complex, non-linear relationships within microbiome data that may be overlooked by traditional statistical techniques. Similarly, Li’s 2023 study employed bidirectional MR to explore causal links between gut microbiota, POAG, and age-related macular degeneration (AMD) [18]. Using genome-wide association studies (GWAS) data, Li et al. provided causal evidence that specific bacterial genera influence IOP—a key risk factor in glaucoma [18]. Building on this approach, Wu et al. further applied MR to confirm causal relationships between microbial taxa and POAG endophenotypes [19]. Their work demonstrated MR’s utility in overcoming confounding limitations in observational studies [19]. Zhou et al. expanded on this approach by combining MR with ML techniques to assess the role of *Lachnospiraceae* in IOP regulation [10]. This integrated approach illustrates how AI-based tools enhance the precision of data-driven models, offering both predictive power and potential pathways for intervention.

These findings underscore the growing interest from descriptive to more predictive and mechanistic approaches in microbiome research. The integration of data-driven and AI-enhanced models improves our ability to explore causal relationships and uncover complex interactions, as shown by Zhou et al.’s use of MR and ML to identify *Lachnospiraceae* as a regulator of IOP in glaucoma. Compared to earlier studies that relied on traditional statistical methods, advanced data-driven models significantly enhance predictive accuracy and facilitate in silico hypothesis testing, bringing the field closer to actionable clinical insights. These results highlight the need for hybrid models that combine data-driven insights with mechanism-based simulations, such as integrating AI-driven microbial analyses with fluid dynamics and solute transport models. Data Integrated AI approaches could improve prediction and intervention strategies, enhancing our understanding of the links between gut dysbiosis and retinal damage.

## 4. Discussion

The complexity of the relationship between the human microbiome and POAG, coupled with the scarcity of data, presents significant challenges for analysis. Mathematical modeling, broadly categorized into data-driven models and mechanism-driven models, has emerged as a valuable tool for studying these interactions [20]. Data-driven models, including classical statistical approaches and more advanced ML techniques enhanced by AI-based tools, are particularly effective for identifying patterns, associations, and causal relationships in large datasets. For instance, ML methods such as MR have uncovered links between microbial taxa, POAG severity, and IOP. These models have greatly advanced our understanding of how gut dysbiosis may influence glaucoma progression. However, data-driven approaches, while powerful, remain primarily descriptive and focus on statistical associations rather than providing mechanistic insights. This limitation highlights the need for mechanism-driven models, which integrate fundamental biological and physical principles to simulate system behavior [20]. These models can offer deeper understanding of causal pathways, such as how microbial metabolites may diffuse through systemic circulation, interact with immune components, and contribute to optic nerve damage. Despite their potential, such approaches have been rarely applied to the study of the gut–eye axis, and the existing literature on this relationship remains limited.

### 4.1. Advantages of AI Modeling

AI offers a powerful computational platform that enhances data-driven models by automating pattern recognition, handling high-dimensional datasets, and optimizing predictive accuracy. These tools can process the vast complexity of microbiome data, uncovering relationships and trends that may be difficult to detect with traditional approaches. Furthermore, AI-based methods enable researchers to simulate scenarios—such as the impact of shifts in microbial communities on immune responses and IOP—through predictive modeling. This simulation approach, often referred to as a Virtual Laboratory (VL) [21], provides a computational environment for testing hypotheses and exploring intervention strategies in silico. The integration of AI tools into data-driven models has transformed microbiome research, accelerating discoveries and providing predictive capabilities that surpass those of purely observational clinical studies. The development of hybrid models that integrate AI-enhanced data-driven frameworks with physics-based mechanistic simulations holds the potential for deeper, more actionable insights into the gut–eye axis.

Incorporating AI-driven data models into the study of the gut–eye axis and POAG offers significant advantages in improving diagnosis and therapeutic strategies. AI’s ability to process complex, high-dimensional datasets allows for more accurate risk stratification, potentially identifying individuals at greater risk for POAG based on their microbiome profiles. This could lead to sooner detection, more targeted screening, and more personalized management plans, particularly for high-risk populations. Furthermore, these hybrid models have the potential to reveal novel therapeutic targets and inform the development of microbiome-based interventions to prevent or slow disease progression. Ultimately, this integration can lead to more targeted, effective treatments that address the underlying causes of POAG, moving beyond symptom management and providing a more holistic approach to care.

### 4.2. Disadvantages of AI Modeling

Despite their advantages, data-driven models, including AI-based methods, are heavily dependent on the quality of input data. Variability in study design, sampling techniques, and sequencing methods can introduce bias, leading to unreliable predictions. Moreover, the heterogeneity of microbiome composition—shaped by factors such as age, diet, and geography—complicates modeling efforts and limits the generalizability of findings across diverse populations. While AI tools excel in identifying patterns and generating predictions, they often suffer from the “black box” problem, which refers to the lack of transparency in how AI makes decisions. This lack of interpretability poses challenges for clinicians who require mechanistic insights to inform clinical decisions.

Mechanism-driven models, though well suited for simulating causal processes, remain underutilized in microbiome-glaucoma research. These models require precise parameterization and well-characterized input data, which are often lacking in microbiome studies. Additionally, the complexity of microbial ecosystems and their interactions with host physiology presents significant computational challenges. The lack of integration between data-driven models and mechanistic-based approaches represents a critical gap. Combining these frameworks, with AI tools serving as a computation bridge, could enable the development of hybrid models that deliver both predictive accuracy and mechanistic insights. Such integration would enhance the ability to understand and address complex interactions within the gut–eye axis. Lastly, the application of AI in clinical settings also raises ethical considerations. Issues such as data privacy, informed consent, and algorithmic bias must be carefully addressed to ensure equitable and transparent clinical applications. The “black box” nature of some AI models may limit their interpretability, potentially undermining clinician and patient trust. Future efforts should prioritize the development of ethical frameworks that ensure fairness, accountability, and transparency in AI-driven diagnostic and therapeutic innovations.

## 5. Future Directions

A key challenge in understanding the role of the gut microbiome in glaucoma is the lack of large, well-controlled longitudinal data. Tracking temporal changes in the gut microbiome of POAG patients is essential for establishing causal relationships between microbiota alterations and disease progression. Future research studies should aim to incorporate physiological data—such as IOP fluctuations, inflammatory markers, and retinal imaging metrics—with microbiome profiles to improve the biological relevance and predictive accuracy of current models. Advanced methodologies, such as genome-wide association studies (GWAS) coupled with MR, can provide insights into causal mechanisms linking microbial communities to glaucoma pathogenesis.

Addressing population diversity is another critical consideration for future research. Many microbiome studies are limited to specific geographic or ethnic cohorts, reducing the generalizability of findings. Including diverse populations in future studies can uncover population-specific microbial markers, reduce biases associated with homogenous datasets, and validate the microbiome’s role in POAG across different demographic groups. Broadening study demographics will help ensure that findings are clinically applicable on a global scale.

A review of the current literature reveals a noticeable gap in mechanism-based modeling approaches within microbiome-glaucoma research. Mechanism-based modes rely on fundamental physical laws, such as those governing fluid dynamics, solute diffusion and transport, and ion electrodiffusion, to provide a mechanistic understanding of biological systems. These models can simulate key processes, such as cellular transport, diffusion of microbial metabolites, and immune response dynamics. By developing mathematical frameworks, mechanism-based approaches are capable of not only explaining associations but, more importantly, unraveling causal interactions within the gut–eye axis that influence glaucoma progression [22].

Unlike data-driven methods which primarily identify statistical associations, mechanism-based models offer mechanistic explanations and predictive capabilities that deepen our understanding of system dynamics. By simulating the behavior of microbial communities and their biochemical interactions over time, these models could enhance the clinical applicability of microbiome research. Incorporating such approaches into microbiome-glaucoma research could clarify underlying mechanisms and push the field from associative studies to actionable insights.

To advance research design, future studies could employ hybrid computational frameworks that integrate AI-enhanced data models with mechanism-based simulations. For instance, stochastic differential equations (SDEs) could model microbial dynamics over time, capturing interactions with host systems such as IOP fluctuations and immune responses. Physics-informed neural networks (PINNs) [23] could combine data with physical laws to improve model accuracy. Additionally, Monte Carlo simulations or Bayesian inference could estimate model parameters from longitudinal datasets. These approaches would not only validate hypotheses but also identify key factors driving disease progression.

The development of hybrid computational frameworks that merge AI-enhanced data-driven models with mechanism-driven simulations represents a promising avenue for future research. These integrative approaches would leverage the strengths of large-scale data analysis and mechanistic modeling to provide a more comprehensive understanding of the gut–eye axis and its role in glaucoma pathogenesis. By bridging the gap between statistical associations and causal mechanisms, such frameworks could yield actionable insights and pave the way for early detection, targeted therapies, and improved clinical outcomes.

## 6. Conclusions

Emerging evidence suggests the existence of a gut–eye axis that influences glaucoma pathogenesis and progression. Although current preliminary data in the literature are limited and highly diverse, advances in data-driven models, enhanced by AI-based tools, have provided valuable insights into associations between gut dysbiosis and glaucoma severity. These approaches excel at identifying patterns and potential causal relationships but often lack the capacity to offer mechanistic explanations.

The integration of mechanism-driven models remains largely unexplored in this context and the authors believe this is a crucial direction for future research. Developing hybrid computational frameworks that combine AI-enhanced data-driven models with physics-based simulations represents a critical next step in advancing our understanding of the gut–eye axis. These approaches can potentially bridge the gap between statistical associations and mechanistic insights.

In conclusion, while the literature on AI and microbiome’s relationship to glaucoma remains limited, understanding this interplay has the potential to transform our knowledge of glaucoma pathogenesis. Future breakthroughs could lead to the identification of novel diagnostic biomarkers and therapeutic strategies, significantly advancing the clinical management of POAG on a global scale.

To achieve this, future research efforts must address challenges related to data quality, incorporate physiological parameters such as IOP fluctuations and inflammatory markers, and improve the representation of diverse populations to enhance the reliability and generalizability of findings. Importantly, these efforts require an interdisciplinary approach that fosters collaboration among clinicians, research scientists, and mathematicians. By leveraging the synergy between data-driven approaches and mechanism-based models supported by AI tools, researchers can achieve a more comprehensive understanding of the microbiome’s role in glaucoma progression. This deeper insight holds the promise of improving patient care and outcomes worldwide.

## Figures and Tables

**Table 1:** Summary of studies exploring the gut–eye axis using classical statistical approaches.

Study	Methodology	Key Findings	Limitations
Chen et al. (2023) [12]	Correlation Analysis, ANOVA	Identified genetic markers associated with POAG.	Limited in addressing complex, non-linear interactions and lacks causal inference capabilities.
Parker et al. (2022) [13]	PCA, Regression Analysis	Demonstrated shifts in gut microbiome composition linked to glaucoma.	Limited in addressing complex, non-linear interactions and lacks causal inference capabilities.
Zysset-Burri et al. (2023) [14]	PCA, Correlation Analysis	Highlighted associations between microbiota and immune responses.	Small sample size. Limited in addressing complex, non-linear interactions and lacks causal inference capabilities.
Vergroesen et al. (2024) [15]	Correlation Analysis	Identify microbiome markers associated with specific POAG phenotypes.	Limited in addressing complex, non-linear interactions and lacks causal inference capabilities.
Astafurov et al. (2014) [16]	Correlation Analysis	Linked microbial taxa to neurodegeneration in POAG.	Limited in addressing complex, non-linear interactions and lacks causal inference capabilities.

**Table 2:** Summary of studies exploring the gut–eye axis using mathematical modeling and AI-based methods.

Study	Methodology	Key Findings	Limitations
Yoon et al. (2021) [17]	Random forest regression	Identified Lactococcus as a potential biomarker for POAG severity.	Limited dataset size; results need validation in larger cohorts. Limited mechanistic insights.
Li et al. (2023) [18]	Bidirectional mendelian randomization (MR)	Found causal links between gut microbiota and intraocular pressure (IOP).	Relies on GWAS data; lacks diversity in population samples. Limited mechanistic insights.
Wu et al. (2024) [19]	Mendelian randomization	Confirmed causal relationships between microbial taxa and glaucoma endophenotypes.	Observational data may introduce confounding variables. Limited mechanistic insights.
Zhou et al. (2024) [10]	Combined MR and Machine Learning	Highlighted Lachnospiraceae’s role in regulating IOP.	Integration of MR and ML requires high-quality input data. Limited mechanistic insights.

## Data Availability

No new data were created or analyzed in this study. Data sharing is not applicable to this article.
